# Group B streptococcus virulence factors associated with different clinical syndromes: Asymptomatic carriage in pregnant women and early-onset disease in the newborn

**DOI:** 10.3389/fmicb.2023.1093288

**Published:** 2023-02-13

**Authors:** Yulia Schindler, Galia Rahav, Israel Nissan, Orit Treygerman, George Prajgrod, Bracha Zukerman Attia, Ronit Raz, Gal Zizelski Valenci, Dorit Tekes-Manova, Yasmin Maor

**Affiliations:** ^1^Laboratory of Microbiology, Mayanei Hayeshua Medical Center, Bnei Brak, Israel; ^2^Sackler School of Medicine, Tel Aviv University, Tel Aviv, Israel; ^3^Infectious Disease Unit, Sheba Medical Center, Tel HaShomer, Israel; ^4^National Public Health Laboratory, Ministry of Health, Tel Aviv, Israel; ^5^Laboratory of Microbiology, Meuhedet Health Maintenance Organization, Lod, Israel; ^6^Infectious Disease Unit, Wolfson Medical Center, Holon, Israel

**Keywords:** group B streptococcus, intra uterine fetal death, Orthodox Jews, antibiotic resistance, *Streptococcus agalactiae*, early-onset sepsis, serotype

## Abstract

**Background:**

Group B streptococcus (GBS) harbors many virulence factors but there is limited data regarding their importance in colonization in pregnancy and early-onset disease (EOD) in the newborn. We hypothesized that colonization and EOD are associated with different distribution and expression of virulence factors.

**Methods:**

We studied 36 GBS EOD and 234 GBS isolates collected during routine screening. Virulence genes (pilus-like structures-*PI-1, PI-2a, PI-2b*; *rib* and *hvgA*) presence and expression were identified by PCR and qRT-PCR. Whole genome sequencing (WGS) and comparative genomic analyses were used to compare coding sequences (CDSs) of colonizing and EOD isolates.

**Results:**

Serotype III (ST17) was significantly associated with EOD and serotype VI (ST1) with colonization. *hvgA* and *rib* genes were more prevalent among EOD isolates (58.3 and 77.8%, respectively; *p* < 0.01). The pilus loci *PI-2b* and *PI-2a* were more prevalent among EOD isolates (61.1%, *p* < 0.01), while the pilus loci *PI-2a* and *PI-1* among colonizing isolates (89.7 and 93.1% vs. 55.6 and 69.4%, *p* < 0.01). qRT PCR analysis revealed that *hvgA* was barely expressed in colonizing isolates, even though the gene was detected. Expression of the *rib* gene and *PI-2b* was two-fold higher in EOD isolates compared to colonizing isolates. Transcription of *PI-2a* was three-fold higher in colonizing isolates compared to EOD isolates. ST17 isolates (associated with EOD) had a smaller genome size compared ST1 and the genome was more conserved relative to the reference strain and ST17 isolates. In a multivariate logistic regression analysis virulence factors independently associated with EOD were serotype 3, and *PI-1* and *PI-2a* was protective.

**Conclusion:**

There was a significant difference in the distribution of *hvg A*, *rib*, and *PI* genes among EOD (serotype III/ST17) and colonizing (serotype VI/ST1) isolates suggesting an association between invasive disease and these virulence factors. Further study is needed to understand the contribution of these genes to GBS virulence.

## Introduction

Group B streptococcus (GBS), also known as *Streptococcus agalactiae*, is a beta-hemolytic, aerobic, Gram-positive, polysaccharide-encapsulated streptococcus. GBS isolates can be divided into ten distinct serotypes (Ia, Ib, and II to IX) based on a serological reaction directed against the polysaccharide capsule (Shabayek and Spellerberg, 2018; Filkins et al., 2021). Multilocus sequence typing (MLST) demonstrates that most human GBS isolates are clustered into six clonal complexes (CCs) (Melin, 2011; Russell et al., 2017; Shabayek and Spellerberg, 2018; Schindler et al., 2020; Filkins et al., 2021). GBS is a commensal bacterium that belongs to the human microbiota colonizing the gastrointestinal and genitourinary tract. GBS colonization in humans is usually not clinically significant but can cause severe diseases, in particular neonatal sepsis, and meningitis (Dermer et al., 2004). Two GBS-associated syndromes are described in neonates: early-onset disease (EOD) and late-onset disease (LOD) (Dermer et al., 2004; Joubrel et al., 2015). EOD, which is mostly associated with bacteremia, occurs in the first week of life (0–6 days), and results from vertical transmission of GBS from the colonized mother through contaminated amniotic or vaginal secretions to her newborn during, or just before delivery. EOD rate varies between 0.3 and 0.6 infants per 1,000 live births (Dermer et al., 2004; Joubrel et al., 2015). The rate of GBS EOD and vaginal colonization varies between geographical areas as do the predominant serotypes (Russell et al., 2017). The most recent report regarding global vaginal GBS colonization estimates a prevalence of 18%, with the lowest prevalence in Southern and Eastern Asia (11–13%) and the highest prevalence in the Caribbean (35%) (Russell et al., 2017; Shabayek and Spellerberg, 2018). Serotypes Ib, II, and V are prevalent colonizers in the United States (US) and Europe (Gherardi et al., 2007; Bergal et al., 2015; Genovese et al., 2020), whereas serotypes VI and VIII are prevalent in Japan (Lachenauer et al., 1999; Kawaguchiya et al., 2022). ST17 isolates mostly belonging to serotype III have been described in some geographical regions as a hypervirulent clone responsible for a large proportion of EOD (Melin, 2011; Teatero et al., 2014; Filkins et al., 2021; McGee et al., 2021).

Since 2017, we have noticed an increase in GBS associated neonatal sepsis and meningitis in Mayanei Hayeshua Medical Center (MHMC), which serves an Orthodox Jewish community in central Israel. The incidence of neonatal EOD disease increased significantly from 0.25/1,000 during 2016 (three cases) to 0.51/1,000 live births (seven cases) in 2017, significantly higher than the national Israeli Ministry of Health GBS annual reported rate of 0.27/1,000 live births. GBS colonization rate in MHMC was significantly higher (26.1%) than the overall prevalence reported among pregnant Israeli women (21.6%) (Data from the Israeli Ministry of Health). In a previous study, we found that the dominant colonizing serotype among asymptomatic pregnant women was serotype VI, while serotype III was the most prevalent among EOD cases. Furthermore, ST-1 was associated with colonization, while ST17 was associated with EOD (Schindler et al., 2020). GBS genome is 2.2 Mbp long and has over 2,100 predicted coding regions. GBS may produce a wide variety of virulence factors, including adhesins that enable penetration of epithelial and endothelial cellular barriers; factors that inhibits immunological clearance, and toxins that directly injure or disrupt host tissue components (Supplementary Appendix Table 1; Rosini et al., 2006; Carreras-Abad et al., 2020). Despite knowledge regarding many virulence factors related to GBS, there is limited information about virulence factors in the context of pregnancy and birth. Here we chose to focus on the more interesting and recently studied virulence factors, pili structures, Rib protein, and the hyper virulent adhesin HvgA, because they are considered important candidates in the development of vaccines against GBS (Carreras-Abad et al., 2020; McGee et al., 2021). Pili promotes colonization of epithelial cell surfaces, support biofilm formation, and facilitate translocation across the blood–brain barrier (Lauer et al., 2005; Dramsi et al., 2006; Rosini et al., 2006). Rib protein belongs to a family of highly repetitive proteins with unique high-molecular-weight in the cell wall, found predominantly in serotype III strain. Rib protein elicits protective immunity in humans and is already being tested in a phase I vaccine study (Fischer et al., 2021; Pulido-Colina et al., 2021). HvgA efficiently supports bacterial adhesion and transfer through to the intestinal wall, and mediates transfer across the blood–brain barrier, specifically the vascular endothelium and the choroid plexus (Tazi et al., 2010). We hypothesized that different clinical syndromes, asymptomatic vaginal colonization during pregnancy, and EOD in the newborns, are related to different distribution of virulence factors.

## Materials and methods

### GBS isolates

The study included a total of 270 GBS isolates (Supplementary Appendix Figure 1 and Supplementary Appendix Tables 2, 3). In a previous study (Schindler et al., 2020) 240 colonizing isolates and 19 EOD isolates were studied. Of these we randomly chose 126 colonizing, and all EOD isolates for further study. To enrich the sample we also obtained 17 additional EOD isolates from (MHMC) and 108 additional colonizing isolates from Meuhedet Health Maintenance Organization, the 3rd largest health plan in Israel (MHMO). Altogether, we studied 36 GBS isolates obtained from blood cultures of neonates with EOD during 2013–2019, and 234 colonizing GBS isolates collected during routine screening of asymptomatic pregnant women at week 35–37 (Supplementary Appendix Figure 1, Supplementary Appendix Tables 2, 3). The study was approved by the Ethics Committee of MHMC (approval number 0023-18-MHMC).

### Bacterial strains and growth conditions

Group B streptococcus isolates were obtained from clinical cultures and preserved in sterile Brain Heart Infusion broth with 15% glycerol (HY-labs, Israel) at −70°C for long-term storage and at 4°C for short-term maintenance.

### DNA extraction

Genomic DNA from GBS isolates was extracted using the EZ1 Virus Mini kit (Qiagen) according to the manufacturer instructions. Frozen GBS strains were revived; twice subcultured on trypticase soy with 5% sheep blood agar plates (TSA, HY-labs) at 37°C with 5% CO_2_. One to three colonies were suspended in one ml of saline. Following centrifugation at 3,200×*g*, the bacteria were lysed in a 15 mg/ml lysozyme solution (Lysozyme, Geneaid). A 400 μL of lysed bacteria were transferred for DNA extraction. Extracted DNA was stored at −20°C.

### Serotyping

All GBS isolates were serotyped using TaqMan real-time PCR. Amplification was performed using specific primers for every serotype and a fluorescent probe labeled with 6-FAM (Breeding et al., 2016). Positive and negative controls were included in every run.

### Virulence gene detection

The presence of genes encoding GBS surface proteins potentially associated with virulence: pili island (*PI-1*, *PI-2a*, *PI-2b*); Rib protein (*rib*) and hypervirulent GBS adhesin (*hvgA*) were evaluated among EOD (*n* = 36), colonizing GBS isolates from MHMC (*n* = 126) and MHMO (*n* = 108). The sets of primers used for detection of virulence genes are listed in the Supplementary Appendix Table 3. The reaction mixture, in final volumes of 50 μL, contained: 10 μL of TaqMan fast advanced master mix (Applied biosystems), 0.2 μL of each primer (10 μM), 7.6 μL nuclease-free PCR water, and 2 μL of DNA. Conditions for amplification were as follows: initial denaturation at 94°C for 3 min, followed by 39 cycles of denaturation at 95°C for 30 s, primer annealing at 55°C for 30 s, and extension at 72°C for 30 s. Amplifications were performed in CFX96 thermocycler (Bio-Rad). In each run a negative control consisting of the reaction mixture with nuclease-free water was added. 10 μL of PCR products were visualized by 2% agarose gel electrophoresis with SYBR Safe. The size of each PCR product was estimated by using standard molecular size markers (100-bp ladder) (GeneDireX, Hylabs). The control GBS isolates were used as positive control and was included to each PCR reaction.

### Genome sequencing and bioinformatics analysis

A sample of GBS isolates were studied: 24 EOD isolates, and 25 colonizing isolates (Supplementary Appendix Table 4). The genomes of GBS isolates were prepared using Nextera XT kits (Illumina, San Diego, CA, USA) and sequenced using the Illumina MiSeq Reagent Kit v3 (600-cycle). Bioinformatics analysis of GBS whole genome sequencing (WGS) was performed by using the bacterial bioinformatics database and analysis resource PATRIC^1^. The reads obtained for each sample were trimmed and the quality of the FASTQ reads was examined using the FASTQ Utilities Service, and finally assembled by SPAdes using the PATRIC website. A high-quality representative genome of *S. agalactiae* 2603 V/R ATCC BAA611 (serotype V, ST19) was used as reference for genomic comparisons (Tettelin et al., 2002; Ondov et al., 2016). The “Phylogenetic Tree Building” service in PATRIC website provided the Codon Tree method that selects a single copy of the amino acid and nucleotide sequences from a defined number of PATRIC’s global Protein Families (PGFams), picked randomly, to build an alignment, and then generate a tree based on the differences within those selected sequences. We performed the protein sequence-based genome comparisons using bidirectional BLASTP by the PATRIC server. This tool provides information about conserved genomic contexts, and the presence of insertions or deletions. We compared the genomic coding sequences (CDSs) of ST17 strains and ST1 strains to the reference strain (Tettelin et al., 2002). The results of representative ST17 and ST1 strains are displayed with color-coding for protein percent identity relative to the best hit on the reference genome. We searched for virulence factors genes by an exhaustive bioinformatic screening of the database “*Virulence Factors Database–VFDB*” (Chen et al., 2005), available at the PATRIC website.

### RNA isolation, reverse transcription, and qRT-PCR

Quantitative qRT-PCR analysis of bacterial gene expression was performed as described previously (Sullivan et al., 2017). Primers were designed using Primer3 Plus software and used at a final concentration of 10 μM (Supplementary Appendix Table 5). The GBS isolates used for this study are listed in the Supplementary Appendix Table 4. RNA was extracted from GBS cultures grown at 37°C to an exponential growth phase in BHI medium. RNA was purified using the RNeasy Mini kit (Qiagen) according to manufacturer instructions. Purified RNA was treated with the DNAse kit (HY-labs, Israel) according to manufacturer instructions. cDNA was synthesized using the Hy-RT-PCR kit (HY-labs, Israel), according to manufacturer instructions. cDNA was diluted 1:150 to further reduce bacterial DNA contamination and qPCR was performed using Hy-SYBR power mix (HY-labs, Israel) and CFX96 Real-Time System (Bio-Rad). RNA from three independent biological triplicates were analyzed and final cycle threshold (Ct) for each strain was calculated (the mean value of three experiments). We compared the expression of tested genes between separate groups (EOD vs. colonizing isolates, ST17 vs. ST1 strains, respectively). For this purpose, the final Ct of each group was determined by calculating the mean CT value of all strains belonging to the group. Relative quantification of gene expression was performed using the comparative 2^–ΔΔ*CT*^ method. The growth rates of colonizing and EOD isolates were tested in BHI medium, and similar growth rates were observed. Results were normalized using *rpoB* gene as the housekeeping gene. Gene expression results of each gene were normalized to its expression.

### Statistical analyses

Means and medians were computed for continuous variables. Number and percent were used to describe categorical variables. Variables were compared using student *T* test or chi square as appropriate. *P*-value was set at < 0.05. For small samples Fisher exact test was used. Mantel–Haenszel common odds ratio estimate was used to calculate 95% confidence intervals.

Multivariate logistic regression (Wald, backward) was performed. The dependent variable was EOD disease. The independent candidate variables were serotypes (3; 6; 1a; 2; 5; 4; other serotypes), and the various virulence factors tested (*PI-2a*; *PI-2b*; *hvgA*; *RIB*; *PI-1*).

### Data availability

The sequences are now available to the public at the NCBI (BioProject number BioProject ID PRJNA861829, at the following link: http://www.ncbi.nlm.nih.gov/bioproject/861829).

## Results

### Serotype distribution

The dominant GBS serotype isolated from colonized asymptomatic pregnant women in MHMO, was serotype VI [32.4% (35/108 isolates)], followed by serotypes III [22.2% (24/108 isolates)], V [(13.9%, 15/108 isolates)], and IV [10.1% (10/108 isolates)] (Table 1). Serotype II was found only in MHMO isolates. The distribution of GBS serotypes in colonizing isolates in MHMO was comparable to the distribution of GBS serotypes in asymptomatic pregnant women in MHMC (*p* > 0.05).

**TABLE 1:** Serotype distribution in colonizing group B streptococcus (GBS) isolates obtained from Meuhedet Health Maintenance Organization (MHMO) compared to colonizing isolates from Mayanei Hayeshua Medical Center (MHMC).

Serotype	No. of GBS isolates tested (%)	
	**MHMO (*N* = 108)**	**MHMC (*N* = 126)**
Ia	7 (6.5%)	5 (3.9%)
Ib	4 (3.7%)	2 (1.6%)
II	12 (11.1%)	0
III	**24 (22.2%)**	**39 (31%)**
IV	10 (10.1%)	18 (14.2%)
V	15 (13.9%)	11 (8.7%)
VI	**35 (32.4%)**	**51 (40.5%)**
VII	1 (0.9%)	0
VIII	0	0
IX	0	0

Serotype distribution in colonizing GBS isolates obtained from Meuhedet Health Maintenance Organization (MHMO), representing the GBS distribution in pregnant Israeli women compared to colonizing isolates from MHMC, that serves mainly a community of Orthodox Jews in Bnei Brak, Central Israel. The serotype distribution in MHMC was comparable to that observed in MHMC, *p* > 0.0, except of serotype III. The most common serotypes in the two centers are marked in bold letters.

### Distribution of virulence factors

**FIGURE 1:**
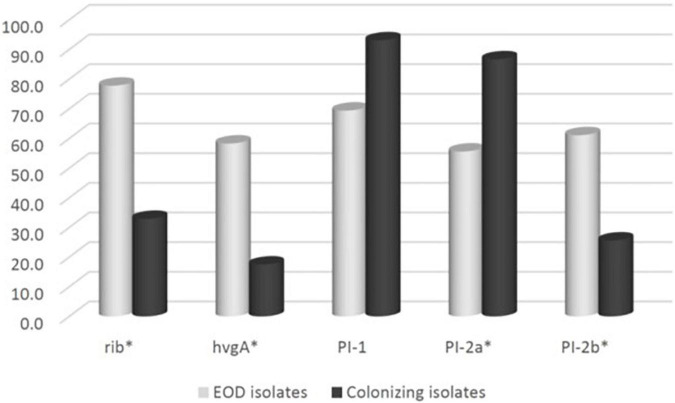
The frequency of virulence genes encoding surface adhesins (*hvgA* and *rib*) and pilus islands (*PI-1*, *PI-2a*, *PI-2b*) among 270 group B streptococcus (GBS) isolates: Early-onset disease (EOD) (*n* = 36) and colonizing isolates (*n* = 234).

We compared the distribution of virulence genes: *rib*, *hvgA*, *PI-1*, *PI-2a*, and *PI-2b* among GBS isolates obtained from pregnant women at MHMC (*n* = 126) and MHMO (*n* = 108). Overall, the virulence gene distribution among both groups was similar (*p* > 0.05) (Table 2), except for *PI-2a* which was less prevalent in MHMC [108/126 (85.7%) vs. 102/108 (94.4%), *p* = 0.028]. The distribution of virulence genes, among isolates obtained from EOD revealed a high prevalence of surface adhesins compared to isolates obtained from asymptomatic pregnant women (*hvgA* and *rib*) (58.3 and 77.8%, respectively; *p* < 0.01) (Figure 1). Pilus loci *PI-2b* was also more prevalent among EOD isolates (61.1% vs. 24.4%; *p* < 0.01), while the pilus loci PI-2a and PI-1 were more frequent among colonizing isolates (89.7 and 93.1% vs. 55.6 and 69.4%; *p* < 0.01) (Figure 1). The distribution of virulence genes among various serotypes was studied (Table 3). Genes encoding for pilus islands *PI-1* and *PI-2a* were detected in all GBS serotypes, while the gene encoding the pilus loci *PI-2b* island was mainly detected in serotype III (64.6%). *hvgA* and *rib* genes were also detected mainly in serotype III (57.3 and 84.4%, respectively). There was a significant association between *hvgA*, *rib*, and *PI-2b* genes with serotype III (*p* < 0.01). To determine the frequency of virulence genes among the different STs, we selected 29 EOD and 39 colonizing GBS isolates from MHMC with known sequence types (STs) (32- ST17 isolates, 16 -ST1 isolates, and 20 other ST types) (Supplementary Appendix Table 4). *hvgA* and *rib* genes were more prevalent among ST17 isolates (75 and 75.1%, respectively) compared to their distribution in all other groups (*p* < 0.01). *PI-2b* island was also more prevalent among ST17 isolates (71.9%, *p* < 0.01). *PI-2a* was highly frequent among ST1 isolates (93.7%), while *PI-1* island had a similar distribution among all genotypes (Table 4).

**TABLE 2:** Distribution of virulence genes among colonizing strains from Mayanei Hayeshua Medical Center (MHMC) and Meuhedet Health Maintenance Organization (MHMO).

	Meuhedet Health Maintenance Organization (MHMO) *n* = 108		Mayanei Hayeshua Medical Center (MHMC) *n* = 126		*P*-value	95% confidence interval
**Virulence genes**	***n***	**(%)**	***n***	**(%)**		
*PI-1*	100	92.6	118	93.7	0.749	0.427, 3.258
*PI-2a*	102	94.4	108	85.7	0.028	0.135, 0.924
*PI-2b*	24	22.2	33	26.2	0.480	0.680, 2.270
*rib*	33	30.6	44	34.9	0.478	0.704, 2.221
*hvgA*	16	14.8	25	19.8	0.313	0.715, 2.832

Distribution of virulence genes: Surface adhesins (*rib* and *hvgA*), and pili structures (*PI-1, PI-2a, PI-2b*) among colonizing group B streptococcus (GBS) isolates from MHMC (*n* = 126) and MHMO (*n* = 108). Overall, virulence gene distribution was similar (*p* > 0.05) except *PI-2a* which was less prevalent in MHMC (*p* < 0.028).

**TABLE 3:** The frequency of genes encoding the surface adhesins (*hvgA* and *rib*) and pilus islands (*PI-1*, *PI-2a*, *PI-2b*) among 270 various group B streptococcus (GBS) serotypes from Mayanei Hayeshua Medical Center (MHMC) and Meuhedet Health Maintenance Organization (MHMO).

Serotypes	*N*^0^ of strains	Genes									
		**PI-2b**		**PI-2a**		**PI-1**		***rib***		***hvgA***	
		***N*** **^0^**	**%**	***N*** **^0^**	**%**	***N*** **^0^**	**%**	***N*** **^0^**	**%**	***N*** **^0^**	**%**
Ia	13	**3**	23.1	**12**	92.3	**10**	76.0	**0**	0	**1**	7.7
Ib	6	**1**	16.7	**5**	83.3	**6**	100	**1**	16.7	**0**	0
II	12	**1**	8.3	**12**	100	**11**	91.7	**3**	25	**0**	0
III	96	**62**	64.6*	**76**	79.2	**82**	85.4	**81**	84.4*	**55**	57.3*
IV	28	**4**	14.3	**26**	92.9	**26**	92.9	**3**	10.7	**0**	0
V	26	**2**	7.7	**21**	80.8	**24**	92.3	**2**	7.7	**1**	3.8
VI	88	**6**	6.8	**77**	87.5	**83**	94.3	**15**	17	**5**	5.7
VII	1	**0**	0	**1**	100	**1**	100	**0**	0	**0**	0
VIII	0	**0**	0	**0**	0	**0**	0	**0**	0	**0**	0
IX	0	**0**	0	**0**	0	**0**	0	**0**	0	**0**	0
Total	**270**	**79**	29.3	**230**	85.2	**243**	90	**105**	38.9	**62**	23

*Chi square *p*-value < 0.05. Distribution of genes encoding the surface adhesins (*hvgA* and *rib*) and pilus islands (*PI-1, PI-2a, PI-2b*) among 270 various GBS serotypes. *PI-2b*, *hvgA*, and *rib* genes were mainly detected in serotype III GBS isolates (64.6, 57.3, and 84.4%, respectively). There was a significant association between *hvgA*, *rib*, and *PI-2b* genes with serotype III (*p* < 0.05). Statistical significant values were highlighted in bold.

**TABLE 4:** Association between sequence type (ST) type and presence of virulence genes among 68 group B streptococcus (GBS) isolates from Mayanei Hayeshua Medical Center (MHMC).

	*rib*	*hvgA*	PI-1	PI-2a	PI-2b	Total
ST17	26 (81.3%)*	24 (75%)*	22 (68.8%)	21 (65.6%)	17 (53.1%)	**32**
ST1	4 (25%)	0	15 (93.7%)	**15 (93.6%)**	7 (73.8%)	**16**
Other STs	14 (73.7%)	2 (5.3%)	19 (100%)	15 (63.2%)	5 (31.6%)	**20**

*Chi square *p*-value < 0.01. Association between ST type and presence of virulence genes was determinated among 68 GBS isolates from MHMC. There was a significant association between *hvgA* and *rib* with ST-17 (*p* < 0.0074, *p* < 0.0001, respectively); *PI-2a* with ST-1 (*p* < 0.008). Statistical significant values were highlighted in bold.

### Genomic analysis of GBS isolates

We assessed genome sequences of 24 EOD isolates and 25 colonizing GBS isolates from MHMC. General features of the GBS genome sequences, including genome size, number of contigs, and guanine-cytosine (GC) content are summarized in Table 5. We identified a correlation between the ST type and genome size. The genome size of ST17 strains, which was mostly associated with EOD, was significantly smaller 2,001,326 bp compared to the genome size of ST1 strains causing asymptomatic colonization 2,100,416 bp (*p* < 0.0001).

**TABLE 5:** Genomic meta-data of 49 group B streptococcus (GBS) strains from Mayanei Hayeshua Medical Center (MHMC) [24 early-onset disease (EOD) and 25 colonizing strains.

Strain	Source	ST type	Genome size (bp)	Mean genome size (bp)	SD	No. of contigs	GC content (%)	CDS
117690	EOD	17	1,952,166	2,001,326	33,038.75	47	35.32	2100
135217	EOD	17	1,954,426			39	35.18	1955
101298	EOD	17	1,956,116			41	35.16	1969
106704	EOD	17	1,958,977			42	35.16	1970
123494	EOD	17	1,959,814			46	35.42	2181
M38567	COL	17	1,976,171			41	35.2	1991
112109	EOD	17	1,992,470			43	35.32	2101
139904	EOD	17	1,992,893			43	35.16	1980
M40268	COL	17	1,998,253			41	35.34	1999
127946	EOD	17	1,998,291			42	35.34	2002
M40200	COL	17	1,998,371			41	35.34	2003
127743	EOD	17	1,998,934			43	35.33	2002
139934	EOD	17	1,999,036			41	35.3	2003
118022	EOD	17	2,001,983			42	35.08	2012
118659	EOD	17	2,011,569			38	35.32	2085
M40158	COL	17	2,014,292			43	35.3	2047
M39881	COL	17	2,015,862			38	35.39	2005
M38345	COL	17	2,015,870			45	35.2	2047
M40376	COL	17	2,016,361			38	35.36	2053
129618	EOD	17	2,022,841			44	35.32	2036
134924	EOD	17	2,027,570			47	35.37	2003
M38603	COL	17	2,030,084			38	35.64	2176
121684	EOD	17	2,039,347			52	35.38	2192
M38291	COL	17	2,100,127			38	35.39	2007
125416	EOD	23	1,909,126	2,100,416	62,369.45	29	35.26	1896
112767	EOD	178	2,012,915			43	35.16	1979
W19655	COL	1	2,035,207			20	35.3	2038
M42204	COL	19	2,042,352			55	35.4	2034
M41387	COL	130	2,052,446			38	35.4	2070
M40064	COL	1	2,056,223			18	35.37	2055
M38421	COL	8	2,061,907			34	35.1	2088
M40042	COL	1	2,083,880			19	35.2	2081
M38742	COL	1	2,092,052			10	35.35	2079
111236	EOD	18	2,092,413			43	35.32	2100
M40083	COL	19	2,094,244			53	35.5	2107
M38307	COL	1	2,096,218			19	35.37	2101
136117	EOD	196	2,110,014			30	35.39	2136
137471	EOD	4	2,127,936			30	35.3	2171
96655	EOD	1	2,128,796			15	35.34	2112
M41827	COL	1	2,129,556			17	35.3	2120
M39135	COL	12	2,129,642			40	35.3	2168
M40942	COL	1	2,130,091			17	352	2118
M39081	COL	1	2,130,695			17	35.3	2123
M38914	COL	1	2,131,011			17	35.3	2123
107488	EOD	1	2,134,184			14	35.33	2124
M38346	COL	19	2,154,193			53	35.45	2183
119317	EOD	27	2,182,713			90	35.45	2248
M42210	COL	27	2,194,420			72	35.4	2255
104854	COL	106	2,198,156			79	35.56	2273

Genomic meta-data of 49 GBS isolates from MHMC (24 EOD and 25 colonizing isolates). The data for each isolate was organized and sorted by genome size (from largest to smaller) and includes ST type, number of contigs, guanine-cytosine content (GC) and coding sequence (CDS). colonizing isolates (COL).

**FIGURE 2:**
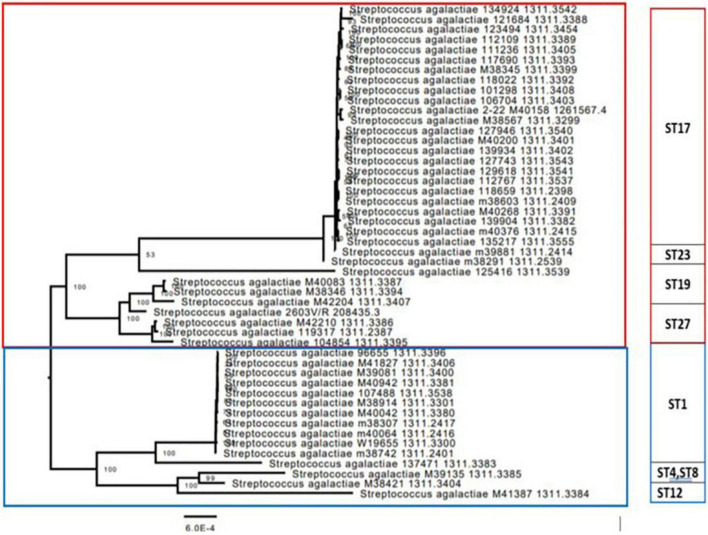
Phylogenetic codon tree (for 1,000 shared genes) of the group B streptococcus (GBS) isolates was built, together with the most closely related ATCC strain of *Streptococcus agalactiae* 2603 V/R (ATCC BAA611). The 49 isolates were clustered into five groups, which corresponded to their multilocus sequence typing (MLST) sequence types (STs). The first branch was mainly composed of ST17, ST19, and ST27 strains, belonging to early-onset disease (EOD) and colonizing isolates, which had very close genetic distance from each other. The second branch was composed from two main clusters mostly containing isolates belonging to colonizing GBS isolates, corresponding to various STs (ST1, ST4, ST8, ST12, and ST130). The phylogenetic distribution of the two branches suggests that the clusters composing these branches belong to divergent lineages.

We constructed a phylogenetic tree of 49 isolates, and a reference strain, to assess the evolutionary relationships among colonizing and EOD GBS isolates. The tree demonstrated five clusters, which corresponded to their MLST STs (Figure 2). The first cluster was mainly composed of ST17, ST19, ST23, and ST27 strains, belonging to EOD and colonizing isolates. The second cluster included mainly colonizing isolates corresponding to various STs (ST1, ST4, ST8, ST12, and ST130). The phylogenetic distribution of the two clusters suggests that they belong to divergent lineages.

**FIGURE 3:**
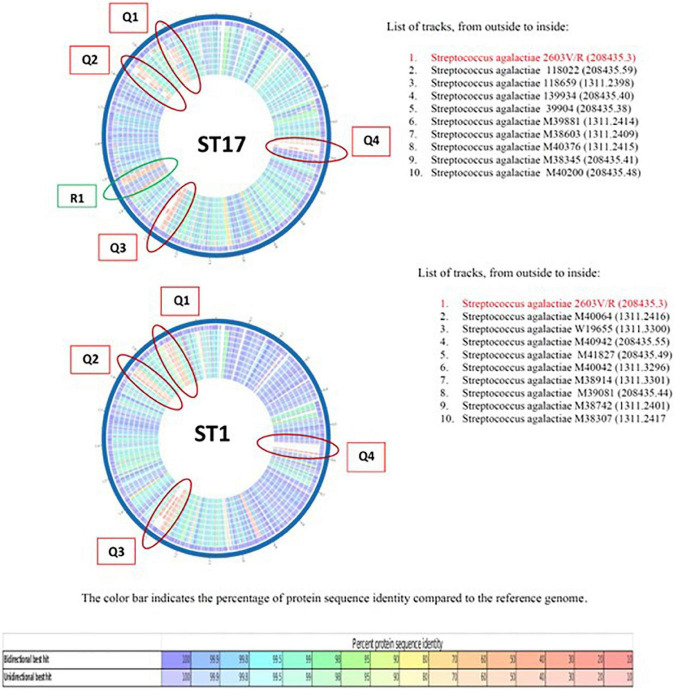
The comparison of representative nine ST17 and nine ST1 strains relative to a reference group B streptococcus (GBS) genome *S. agalactiae* 2603 V/R (ATCC BAA611). Colored vs. white highlights insertions/deletions. Changes in conservation relative to the reference genome (going from blue representing the highest protein sequence similarity to red representing the lowest). Each circle represents the genome of GBS isolate.

To study further the genetic differences between EOD (ST17) and colonizing (ST1) GBS isolates we performed protein sequence-based genome comparisons using bidirectional BLASTP by the PATRIC server. This tool provides information about conserved genomic contexts, and the presence of insertions or deletions. The results of representative nine ST17 and nine ST1 strains are displayed in Figure 3, demonstrate different clusters of conserved genes between the two ST types. There were significant differences in gene sequences between ST17 and ST1 strains. ST1 strains showed a conserved genome relative to the reference strain (>99.9% protein sequence identity), while ST17 strains had less similarity (≤98%). Four mutant gene islands were characterized in both ST17 and ST1 strains (Q1–Q4), compared to the reference strain. In ST17 strains, Q3 and Q4 regions contained both gene deletions and point mutations, while in ST1 strains these regions contained only point mutations. The sequences in these mutant gene islands were mainly coded for phage-related proteins, hypothetical proteins with unknown function, and gene recombination related enzymes. ST17 isolates also carried one mutation-enriched region (Y1), that contained point mutations with sequence identity below 50%. Most of the genes in this region were associated with metabolism and transport (Supplementary Appendix Table 6).

### Virulence factors

To further characterize the presence and role of virulence genes in GBS isolates, we performed further analyses in 49 sequenced GBS isolates from MHMC by employing the database *VFDB* using the PATRIC website. All GBS isolates tested shared the following virulence factors, which contribute to adhesion (*lmb*, *hylB*), protease activity (*scpB*), biofilm formation (*hasC*), and toxins production (*cfa*, *cyl*) (Table 6). The main differences between colonizing and EOD isolates were found regarding the Rib surface protein (*rib*) and fibrinogen binding protein type B (*fbsB*). These proteins were widespread among EOD isolates and rarely identified among colonizing isolates. Pilus island-2 (PI-2) was frequently identified among colonizing isolates (genes encoded for the backbone pilin protein, *PilA* and *PilC* were found in 80% of colonizing isolates). *PI-1* had the same distribution across all the isolates regardless of clinical presentation. PI-2 was not detected by this approach.

**TABLE 6:** Percent of isolates positive for virulence genes across 49 group B streptococcus (GBS) strains from Mayanei Hayeshua Medical Center (MHMC) (detected by PATRIC based on *VGFD* database).

Category	Virulence gene	Gene	EOD strains	Colonizing strains
			***N*** ** = 24**	***N*** ** = 25**
Adherence	**Fibrinogen binding protein**	***fbsA***	12.5	28
		***fbsB***	75	32
	**Laminin binding protein**	***lmb***	95.8	100
	**Pilus island I**	***gbs0628***	62.5	96
		***gbs0630***	62.5	96
		***gbs0631***	62.5	96
		***gbs0632***	100	100
	**Pilus island II**	***pilA***	25	80
		***pilB***	0	8
		***pilC***	25	80
		***srtC3***	20.8	75
		***srtC4***	20.8	75
	**Hyaluronidase**	***hylB***	100	96
Immune evasion	**Capsule biosynthesis**	***neuA-D***	100	100
Immunoreactive antigen	**Surface protein Rib**	***rib***	88.8	50
Protease	**C5a peptidase**	***scpB***	95.8	100
Toxin	**CAMP factor**	***cfa***	100	100
	**Beta-hemolysin/cytolysin**	cyl operon*	100	100
Biofilm formation	**Hyaluronic acid capsule**	***hasC***	10	8

*The cyl operon is made up of 12 genes (*cylX, cylD, cylG, acpC, cylZ, cylA, cylB, cylE, cylF, cylI, cylJ, cylK*).

### Expression profiling of virulence genes in EOD and colonizing GBS isolates

**FIGURE 4:**
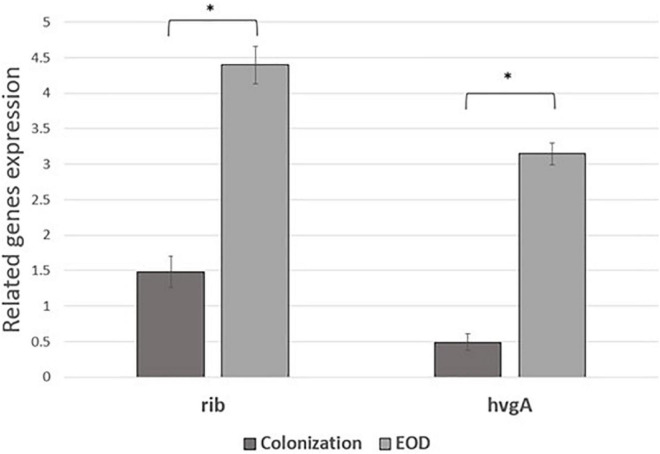
The expression of rib and *hvgA* gene expression in early-onset disease (EOD) and colonizing isolates from Mayanei Hayeshua Medical Center (MHMC) were analyzed by qRT-PCR. Amounts of each transcript were normalized to *rpoB* gene and expressed relative to this gene in reference strain ATCC BAA611. The expression level of ATCC BAA611 strain was defined as 1. Columns represent the relative mRNA expression level of each gene from each strain. Values are represented as means ± SD (*n* = 8) from three independent qRT-PCR experiments. Error bars show SD. Asterisk indicates a significant difference (*p < 0.05 by *t*-test).

**FIGURE 5:**
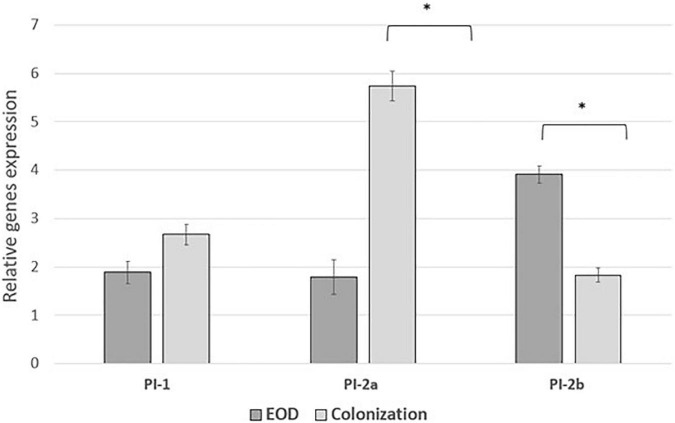
The expression of pili islands gene in early-onset disease (EOD) and colonizing group B streptococcus (GBS) isolates from Mayanei Hayeshua Medical Center (MHMC) were analyzed by qRT-PCR. Amounts of each transcript were normalized to the *rpoB* gene and expressed relative to this gene in reference strain ATCC BAA611. The expression level of ATCC BAA611 strain was defined as 1. Columns represent the relative mRNA expression level of each gene from each strain. Values are represented as mean ± SD (*n* = 8) from three independent qRT-PCR experiments. Error bar show SD. Asterisk indicates a significant difference (*p < 0.05 by *t*-test).

**FIGURE 6:**
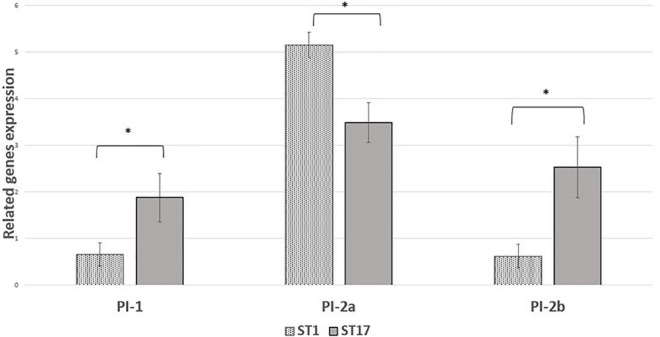
The expression of pili islands gene expression in ST17 and ST1 group B streptococcus (GBS) strains from Mayanei Hayeshua Medical Center (MHMC) were analyzed by qRT-PCR analysis. Amounts of each transcript are normalized to *rpoB* gene and expressed relative to this gene in reference strain ATCC BAA611. The expression level of ATCC BAA611 strain was defined as 1. Columns represent relative mRNA expression level of each gene from each strain. Values are represented as means ± SD (*n* = 8) from three independent qRT-PCR experiments. Error bar show SD. Asterisk indicates a significant difference (*p < 0.05 by *t*-test).

We performed comparative experiments using quantitative PCR for the expression of the virulence genes for EOD (*n* = 8) and colonizing (*n* = 8) isolates. We found that the differences between EOD and colonizing isolates is not only reflected by the presence of virulence genes, but also by their expression (Figure 4). *hvgA* was barely expressed in colonizing ST17 strains, although the presence of this gene was confirmed by PCR. The expression of the *rib* gene was two-fold higher in EOD isolates compared to colonizing isolates. Transcription of *PI-2a* was about three-fold higher in colonizing isolates as compared to EOD isolates. In contrast, the transcription of *PI-2b* was two-fold higher in EOD compared to colonizing isolates (Figure 5). This suggests difference in the regulation of the pilus loci in different GBS isolates mainly those responsible for invasive (EOD) or non-invasive (colonizing isolates) disease. We also investigated the correlation between the expression levels of *PI-2a*, *PI-2b*, and *PI-1* genes in ST17 compared to ST1 strains. RT-qPCR analysis was performed with six ST17 and six ST1 GBS strains. Transcription of *PI-2a* was about two-fold higher in ST1 strains as compared to ST17 strain, while the transcription of *PI-2b* was two-fold higher in ST17 compared to ST1 strains (Figure 6).

In a multivariate logistic regression analysis *PI-I*, *PI-2A*, and serotype 3 were independently associated with EOD (Table 7). The *r*^2^ was 0.511.

**TABLE 7:** Multivariate logistic regression analysis of predictors of early-onset disease (EOD).

Variable	Exp (B)	*P*-value	95% confidence interval
PI-1	0.131	<0.001	0.39–0.439
PI-2A	0.109	<0.001	0.038–0.321
Serotype 3	33.027	<0.001	8.955–121.812
Constant	15.818	<0.001	

Multivariate logistic regression (Wald backward). The dependent variable was EOD. The candidate variables were serotype 3, 6, 2, 5, 4, all other serotypes, *PI-2a, PI-2b, hvgA*, and *RIB*. Nagelkerke R^2^ was 0.511.

## Discussion

We studied aspects of GBS virulence related to molecular differences between GBS isolates derived from different clinical syndromes, vaginal colonization during pregnancy (non-invasive strains), and neonates with EOD (invasive strains). In general, ST17 GBS strains are overrepresented in EOD on the newborn, and considered as hypervirulent, while ST1 strains were associated with asymptomatic carriage during pregnancy (Shabayek and Spellerberg, 2018; Bobadilla et al., 2021).

Evidence of virulent genes distribution is mainly associated with a geographic area of the countries and there is less information regarding the association with specific clinical syndromes (Bobadilla et al., 2021).

In our data, there were significant differences in the distribution of virulence genes between EOD/ST17 GBS isolates and ST1 GBS isolates from colonized pregnant women. Genes encoding for surface adhesins (*hvgA* and *rib*) were more prevalent among EOD isolates compared to their distribution among colonizing isolates (58.3, 77.8%, respectively). These results are consistent with other studies (Brzychczy-Włoch et al., 2012; Campisi et al., 2016; McGee et al., 2021). HvgA is a hypervirulent adhesin suggested to promote meningeal tropism in neonates when the Rib protein confers protective immunity. Both these surface-anchored proteins, act not only as a bacterial adhesins, but can also penetrate the intestinal and blood-brain barriers thus allowing the migration of GBS into the circulatory and central nervous systems (Pereira et al., 2018; Fischer et al., 2021; Pulido-Colina et al., 2021). Our findings demonstrated also that the surface adhesins HvgA and Rib were associated mainly with serotypes III, strongly associated with EOD. Previous data from Southeast Asian countries and Europe supports this association (Lohrmann et al., 2020; Pulido-Colina et al., 2021).

Interestingly, the genes encoding for pilus loci *PI-2b* were more prevalent among EOD isolates, while the genes encoding for pilus loci *PI-2a* and *PI-1* were more prevalent among colonizing isolates. These pilus loci mediate interaction with host cells, involved in bacterial invasion and paracellular translocation mediating resistance to phagocytic killing and virulence (Pezzicoli et al., 2008; Pietrocola et al., 2018). Previous studies where *in vitro* models of GBS infection were involved, have shown that PI-2a was important for biofilm formation (Mandlik et al., 2008; Rinaudo et al., 2010), while PI-2b protein increased intracellular survival in macrophages (Pezzicoli et al., 2008; Motallebirad et al., 2021). Another research demonstrated that PI-1 and PI-2a islands were the most frequently detected surface proteins (88.2 and 82%, respectively) and were found in all tested serotypes (Motallebirad et al., 2021). Our findings regarding virulence gene distribution were not unique to isolates obtained from MHMC, as a similar distribution was found among isolates obtained from MHMO, representing the Israeli pregnant population. The importance of detecting virulence genes with respect to predicting the invasiveness of strains is contradictory. Smith et al. (2007) and Eskandarian et al. (2015) could not demonstrate any correlation between the virulence genes and clinical status of the patients from whom the isolates were obtained. In contrast, Manning et al. (2006) found that invasive strains were associated with specific serotype/gene combinations, but the association was only marginally significant. It is possible that the differences in pathogenicity are not directly related to the virulence genes, but to differences in their expression. RT qPCR analysis revealed that *hvgA* was not expressed in colonizing isolates, even though the presence of gene was detected. The expression of the *rib* gene was two-fold higher in EOD isolates compared to colonizing isolates. Transcription of *PI-2a* was about three-fold higher in colonizing isolates as compared to EOD isolates. In contrast, the transcription of *PI-2b* was two-fold higher in EOD compared to colonizing isolates. These differences in the levels of transcription suggests a difference in the regulation of pilus loci expression in different GBS isolates according to GBS type and clinical illness: invasive (EOD isolates) or colonizing isolates.

This is the first report describing that the genome of ST17 strains is smaller than the ST1 genome. This may be due to evolutionary processes, such as genome size reduction (Didelot et al., 2016; Gatt and Margalit, 2021; Grote and Earl, 2022). Bacterial genome size is mainly determined by gains or losses of genes (Gressmann et al., 2005). Bacteria acquire new genes through duplication of genes or horizontal gene transfer (Moran, 2002; Furuta et al., 2011). Genes may be deleted through mutations or recombination events (Furuta et al., 2011). Being small comes with some advantages, such as needing fewer resources or having more opportunities to hide or escape from predators. Genome reduction occurs when gene losses prevail over gene gains (Björkholm et al., 2001; Moran, 2002; Gressmann et al., 2005). ST17 lineage is derived from a bovine GBS ancestor (Sørensen et al., 2019). Theoretically, it is possible that due to neutral drift among genes that are no longer needed, ST17 strains became more restricted to humans. The same process was identified in Paratyphi A and Typhi, human restricted serovars of *Salmonella enterica* (McClelland et al., 2004). Recently, Murray et al. (2021) published a systematic study of the claim that pathogenicity of various bacteria is associated with genome reduction and gene loss. However, there is no data about a correlation between genome reduction and GBS pathogenesis.

The phylogenetic analysis of GBS isolates revealed two main clusters. Cluster 1: EOD isolates (ST17, ST23, ST19, and ST27) and cluster 2: colonizing isolates (ST1, ST4, ST8, and ST12), which mean that colonizing (ST1) and EOD (ST17) isolates belong to different evolutionary branches with unique evolutionary characteristics. The phylogenetic distribution of two branches supports the MLST findings that clusters composing these branches are not close to each other and belong to divergent lineages. Additionally, we also performed a genomic sequence alignment between ST1 and ST17 strains. Our results identified significant differences in gene sequences between them. The genome of GBS ST1 strains were more conserved compared to the genome of ST17 strains. In both genomes mutant gene islands were identified, but only in ST17 strains an additional mutation enriched region was detected, mainly coded for metabolism and transport related genes. Previous studies have found that genome recombination was a major driver for GBS genetic diversity (Brochet et al., 2006), which can explain the hypervirulence feature of ST17 clones, their possibility to colonize and invade different host tissues.

Vaccination is one of the strategies most likely to be implemented to prevent GBS infections. For designing GBS vaccines, knowledge of the prevalence of immunization targets such as capsular serotypes and alternative targets, such as virulence genes is essential (Bobadilla et al., 2021). In our recent study, we reported that serotype VI (ST1) was a dominant strain among colonizing GBS isolates, however, the serotype III (ST17) strains were significantly associated with EOD. This data could be of interest in the perspective of a future vaccine (Russell et al., 2017).

Our study has several limitations. We performed gene expression tests in a laboratory setting. Although we did our best to standardize tests, still differences of gene expression may exist between *in vitro* and *in vivo* models due to differences in the environmental conditions between *in vivo* and *in vitro* studies. The sample selection process may also introduce biases in the interpretation of the results. To conclude, this study provides information on the distribution of virulence genes among colonizing and invasive GBS strains in Israel. We demonstrated significant differences in the distribution of *hvg A*, *rib*, and *PI-2b* genes among EOD (serotype III/ST17) and colonizing (serotype VI/ST1) isolates thus suggesting an association between virulence and clinical syndromes. Additional studies are needed to investigate these virulence factors involved in immune evasion, host–cell interactions, and successful environmental persistence mechanisms such as biofilm formation. This data can help to define better interventional programs such as vaccine development and preventive therapeutic targets against the GBS.

## Data availability statement

The datasets presented in this study can be found in online repositories. The names of the repository/repositories and accession number(s) can be found below: BioProject ID PRJNA861829.

## Ethics statement

The studies involving human participants were reviewed and approved by the Mayanei Hayeshua Medical Center (approval number: 0023-18-MHMC). Written informed consent for participation was not required for this study in accordance with the national legislation and the institutional requirements.

## Author contributions

YS participated in developing the idea for this work, performed the genetic analyses, and wrote the Introduction, Methods, and Results of the manuscript. IN, GP, BA, RR, and GV performed some of the molecular work and edited the manuscript. GR and DT-M developed the idea, reviewed and edited the manuscript, and contributed to the funding. YM developed the idea, wrote the Discussion, and reviewed and edited the manuscript. All authors contributed to the article and approved the submitted version.

## Footnotes

1
https://www.patricbrc.org/
