# Supplementary material for: Group B streptococcus virulence factors associated with different clinical syndromes: Asymptomatic carriage in pregnant women and early-onset disease in the newborn
Source: Front Microbiol. 2023 Feb 13;14:1093288. doi: 10.3389/fmicb.2023.1093288 (PMC9968972; doi:10.3389/fmicb.2023.1093288)
Supplement: Supplementary file 1 [file Data_Sheet_1.docx]

**Supplementary material**

**Table S1:** The main known virulence factors of GBS

| **Function** | **Virulence factors** | **Gene** | **Molecular or cellular actions** |
| --- | --- | --- | --- |
| Adherence | Fibrinogen binding protein | *fbsA* | Protects GBS from opsono-phagocytosis, promotes adhesion to the epithelial cells and assists in crossing the blood-brain barrier leading to meningitis |
|  | Laminine binding protein | *lmb* | Participates in adhesion to laminin in the extracellular matrix of the host cell |
|  | Hyaluronidase | *hylB* | Cleaves hyaluronan and promotes spreading through host tissues |
| Immune invasion | Capsule | *cps* | Prevents the elimination of GBS by the immune system mainly by preventing deposit of complement, and phagocytosis |
| Immunoreactive antigens | Alpha C protein | *bca* | Facilates GBS adherence and promotes invasion of epithelial cells |
|  | Beta C protein | *bac* | Interferes with IgA effector function and regulates of complement activation |
| Proteases | C5a peptidase | *scpB* | Inactivates human C5a (a chemotactic protein) and inhibits recruitment of neutrophils, and reduces the inflammatory response of the host. |
| Toxins | CAMP factor | *cfb* | CAMP reaction (co-hemolysin), bindes to Fc portion of IgG, IgG |
|  | Beta-hemolysin/cytolysin | *cyl* | Forms pors in cell membranes induces apoptosis promotes cellular evasion |

**Table S2: Serotype distribution in the various analyses**

|  | **Serotyping** | | **MLST** | | **Virulence factors (PCR)** | | **Expression (qPCR)** | | **WGS** | |
| --- | --- | --- | --- | --- | --- | --- | --- | --- | --- | --- |
| **Serotype** | **EOD** | **Colonizing** | **EOD** | **Colonizing** | **EOD** | **Colonizing** | **EOD** | **Colonizing** | **EOD** | **Colonizing** |
| **N** | 36 | 234 | 29 | 39 | 36 | 234 | 8 | 8 | 24 | 25 |
| **Ia** | 1  (2.8) | 12  (5.1) | 1  (3.4) | 0 | 1  (2.8) | 12  (5.1) | 0 | 0 | 0 | 0 |
| **Ib** | 0 | 6  )2.6) | 0 | 0 | 0 | 6  )2.6) | 0 | 0 | 0 | 0 |
| **II** | 0 | 12  (5.1) | 0 | 0 | 0 | 12  (5.1) | 0 | 0 | 0 | 0 |
| **III** | 33  (91.7) | 63  (26.9) | 26  (89.7) | 15  (38.4) | 33  (91.7) | 63  (26.9) | 6  (75.0) | 4  (50.0) | 22  (91.7) | 11  (44.0) |
| **IV** | 0 | 28  (12.0) | 0 | 1  (2.6) | 0 | 28  (12.0) | 0 | 0 | 0 | 0 |
| **V** | 0 | 26  (11.1) | 0 | 0 | 0 | 26  (11.1) | 0 | 0 | 0 | 0 |
| **VI** | 2  (5.6) | 86  (36.8) | 2  (6.9) | 23  (59.0) | 2  (5.6) | 86  (36.8) | 2  (25.0) | 4  (50.0) | 2  (8.3) | 14  (56.0) |
| **VII** | 0 | 1  (0.4) | 0 | 0 | 0 | 1  (0.4) | 0 | 0 | 0 | 0 |
| **VIII** | 0 | 0 | 0 | 0 | 0 | 0 | 0 | 0 | 0 | 0 |
| **IX** | 0 | 0 | 0 | 0 | 0 | 0 | 0 | 0 | 0 | 0 |

**Table S2:** List of primer used for identification of GBS putative virulence gens

| **Amplicon size** | **Sequence (5’-3’)** | **Direction** | **Primer name** | **Gene name** |
| --- | --- | --- | --- | --- |
| 369 bp | CAGGAAGTGCTGTTACGTTAAAC | **F** | rib | ***rib*** |
|  | CGTCCCATTTAGGGTTCTTTCC | **R** | rib |  |
| 210 bp | ATACAAATTCTGCTGACTACCG | **F** | ST-17S | ***hvg1*** |
|  | TTAAATCCTTCCTGACCATTCC | **R** | ST-17AS |  |
| 243 bp | AACTCCCTATATTTGCAGGTTCAA | **F** | Sag1406 | **PI-2a** |
|  | CGGGTGTAACGACTTTTATCTGAT | **R** |  |  |
| 519 bp | GGGGGTAGGCTTAATGGCTTAT | **F** | San1517 | **PI-2b** |
|  | TCCGGTTTAACTGTTCTGATTTGAT | **R** |  |  |
| 394 bp | CTACCAACGGCCAAGCTATTTACC | **F** | Sag647 | **PI-1** |
|  | TAGCCGCTTTTTCATTCTTTCTCC | **R** |  |  |

**Table S3: The list of 49 GBS isolates used in this study**

| **Accession number** | **Specimen type** | **Serotype** | **Sequence type** | **Clonal complex** | **qRT-PCR analysis** |
| --- | --- | --- | --- | --- | --- |
| 99655 | EOD | 6 | ST1 | CC1 | done |
| 101298 | EOD | 3 | ST17 | CC17 |  |
| 104854 | EOD | 3 | ST106 | ST106 |  |
| 106704 | EOD | 3 | ST17 | CC17 |  |
| 107488 | EOD | 6 | ST1 | CC1 | done |
| 111236 | EOD | 3 | ST17 | CC17 |  |
| 112109 | EOD | 3 | ST17 | CC17 |  |
| 112767 | EOD | 3 | ST17 | CC17 |  |
| 117690 | EOD | 3 | ST17 | CC17 |  |
| 118022 | EOD | 3 | ST17 | CC17 |  |
| 118659 | EOD | 3 | ST17 | CC17 | done |
| 119317 | EOD | 3 | ST27 | CC17 | done |
| 121684 | EOD | 3 | ST17 | CC17 |  |
| 123494 | EOD | 3 | ST17 | CC17 | done |
| 125416 | EOD | 3 | ST23 | CC23 | done |
| 127743 | EOD | 3 | ST17 | CC17 |  |
| 127946 | EOD | 3 | ST17 | CC17 | done |
| 129618 | EOD | 3 | ST17 | CC17 |  |
| 134924 | EOD | 3 | ST17 | CC17 |  |
| 135217 | EOD | 3 | ST17 | CC17 | done |
| 136117 | EOD | 3 | ST196 | CC459 |  |
| 137471 | EOD | 3 | ST4 | CC3 |  |
| 139904 | EOD | 3 | ST17 | CC17 |  |
| 139934 | EOD | 3 | ST17 | CC17 |  |
| M38291 | colonization | 3 | ST17 | CC17 | done |
| M38307 | colonization | 6 | ST1 | CC1 | done |
| M38345 | colonization | 3 | ST17 | CC17 |  |
| M38346 | colonization | 3 | ST19 | CC17 |  |
| M38421 | colonization | 6 | ST8 | CC12 |  |
| M38567 | colonization | 6 | ST17 | CC17 |  |
| M38603 | colonization | 3 | ST17 | CC17 |  |
| M38742 | colonization | 6 | ST1 | CC1 | done |
| M38914 | colonization | 6 | ST1 | CC1 |  |
| M39081 | colonization | 6 | ST1 | CC1 | done |
| M39135 | colonization | 3 | ST12 | CC12 |  |
| M39881 | colonization | 6 | ST17 | CC17 |  |
| M40042 | colonization | 3 | ST1 | CC1 | done |
| M40064 | colonization | 6 | ST1 | CC1 |  |
| M40083 | colonization | 3 | ST19 | CC17 | done |
| M40158 | colonization | 3 | ST17 | CC17 | done |
| M40200 | colonization | 3 | ST17 | CC17 |  |
| M40268 | colonization | 3 | ST17 | CC17 |  |
| M40376 | colonization | 3 | ST17 | CC17 |  |
| M40942 | colonization* | 6 | ST1 | CC1 |  |
| M41387 | colonization | 6 | ST130 | Singleton |  |
| M41827 | colonization* | 6 | ST1 | CC1 |  |
| M42204 | colonization | 6 | ST19 | CC17 |  |
| M42210 | colonization | 6 | ST27 | CC17 | done |
| W19655 | colonization* | 6 | ST1 | CC1 |  |

* The isolates used for protein comparisons

** The isolates used for qRT-PCR analysis

**Table S4:** List of primer used for RT-qPCR of virulence gens

| Sequence (5’-3’) | Direction | Primer name | Gene name |
| --- | --- | --- | --- |
| CCTCATACGAAGCCTGATGG | F | rib | *rib* |
| CCTCGTCCCATTTAGGGTCT | R |  |  |
| ATACAAATTCTGCTGACTACCG | F | ST-17S | *hvgA* |
| TTAAATCCTTCCTGACCATTCC | R | ST-17AS |  |
| AATTAGTCCGTTCTCCTGGTGTTT | F | rpoB | rpoB |
| ACGTGTGCGGTCGATACGT | R |  |  |

**Table S5:** Proteins encoded by genes from mutation-enriched region R1 with lowest protein similarity compared to reference genome, in ST17 strains

|  | **Protein name** | **Percent Identity** |
| --- | --- | --- |
| R1 | GftB: Glycosyl transferase, family 8 | 41.3 |
|  | Poly(glycerol-phosphate) alpha-glucosyltransferase GftA (EC 2.4.1.52) | 52.3 |
|  | Protein translocase subunit SecA clustered with accessory secretion system | 51 |
|  | Accessory secretory protein Asp3 | 35 |
|  | Accessory secretory protein Asp2 | 40.4 |
|  | Accessory secretory protein Asp1 | 40.4 |
|  | Protein translocase subunit SecY clustered with accessory secretion system | 29 |
|  | Glycosyl transferase, family 8 | 53.1 |
|  | Beta-1,3-glucosyltransferase | 37.2 |
|  | Glycosyl transferase, family 8 | 40 |
|  | Glycosyl transferase, family 8 | 35.1 |
|  | Glycosyl transferase, family 8 | 37.5 |
|  | Nucleotide sugar synthetase-like protein | 40 |
|  | Predicted cell-wall-anchored protein SasA (LPXTG motif) | 38.2 |

**Table S6.** Distribution of CCs between invasive and colonizing GBS isolates

| **Clonal complex** | **Colonization** | **EOD** | **Total** |
| --- | --- | --- | --- |
| 1 | 9 | 2 | 11 |
| 12 | 2 |  | 2 |
| 17 | 9 | 17 | 26 |
| 19 | 4 | 1 | 5 |
| 23 |  | 1 | 1 |
| 106 |  | 1 | 1 |
| 459 |  | 1 | 1 |
| SINGLETON | 1 | 1 | 2 |
| **Total** | **25** | **24** | **49** |
