# Supplementary figures and images for: Group B streptococcus virulence factors associated with different clinical syndromes: Asymptomatic carriage in pregnant women and early-onset disease in the newborn
Source: Front Microbiol. 2023 Feb 13;14:1093288. doi: 10.3389/fmicb.2023.1093288 (PMC9968972; doi:10.3389/fmicb.2023.1093288)

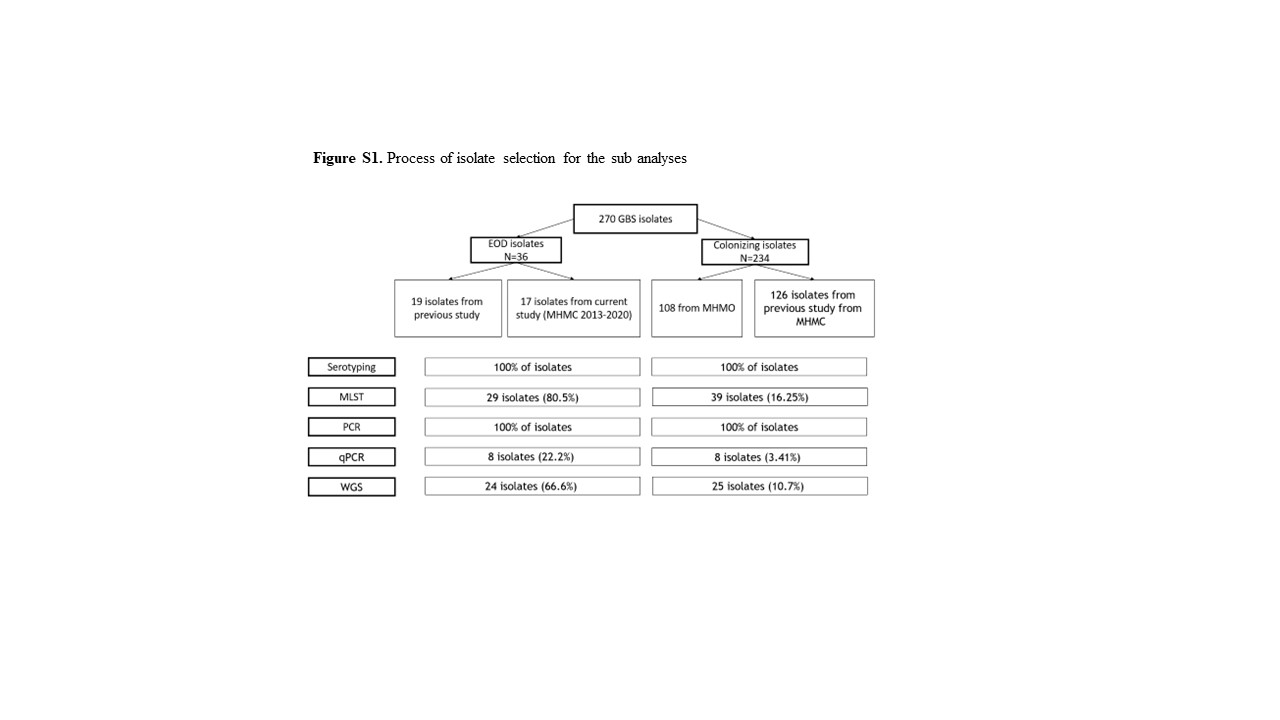

Supplement: Supplementary file 2 [file Image_1.jpg]
